# A community-driven reconstruction of the *Aspergillus niger* metabolic network

**DOI:** 10.1186/s40694-018-0060-7

**Published:** 2018-09-26

**Authors:** Julian Brandl, Maria Victoria Aguilar-Pontes, Paul Schäpe, Anders Noerregaard, Mikko Arvas, Arthur F. J. Ram, Vera Meyer, Adrian Tsang, Ronald P. de Vries, Mikael R. Andersen

**Affiliations:** 1 Technical University of Denmark, Soeltofts Plads, Building 223, 2800 Kongens Lyngby, Denmark; 2 Fungal Physiology, Westerdijk Fungal Biodiversity Institute and Fungal Molecular Physiology, Utrecht University, Uppsalalaan 8, 3584 CT Utrecht, The Netherlands; 3 VTT Technical Research Centre of Finland, Tietotie 2, 02044 Espoo, Finland; 4 Concordia University, 7141 Sherbrooke Street West, H4B1R6 Montreal, Québec Canada; 5 Leiden University, Sylviusweg 72, 2333 BE Leiden, The Netherlands; 6 Berlin University of Technology, Gustav-Meyer-Allee 25, 13355 Berlin, Germany; 7 Present Address: Finnish Red Cross Blood Service, Helsinki, Finland

**Keywords:** *Aspergillus niger*, Primary metabolism, Secondary metabolism, Genome-scale model

## Abstract

**Background:**

*Aspergillus niger* is an important fungus used in industrial applications for enzyme and acid production. To enable rational metabolic engineering of the species, available information can be collected and integrated in a genome-scale model to devise strategies for improving its performance as a host organism.

**Results:**

In this paper, we update an existing model of *A. niger* metabolism to include the information collected from 876 publications, thereby expanding the coverage of the model by 940 reactions, 777 metabolites and 454 genes. In the presented consensus genome-scale model of *A. niger* iJB1325 , we integrated experimental data from publications and patents, as well as our own experiments, into a consistent network. This information has been included in a standardized way, allowing for automated testing and continuous improvements in the future. This repository of experimental data allowed the definition of 471 individual test cases, of which the model complies with 373 of them. We further re-analyzed existing transcriptomics and quantitative physiology data to gain new insights on metabolism. Additionally, the model contains 3482 checks on the model structure, thereby representing the best validated genome-scale model on *A. niger* developed until now. Strain-specific model versions for strains ATCC 1015 and CBS 513.88 have been created containing all data used for model building, thereby allowing users to adopt the models and check the updated version against the experimental data. The resulting model is compliant with the SBML standard and therefore enables users to easily simulate it using their preferred software solution.

**Conclusion:**

Experimental data on most organisms are scattered across hundreds of publications and several repositories.To allow for a systems level understanding of metabolism, the data must be integrated in a consistent knowledge network. The *A. niger* iJB1325 model presented here integrates the available data into a highly curated genome-scale model to facilitate the simulation of flux distributions, as well as the interpretation of other genome-scale data by providing the metabolic context.

**Electronic supplementary material:**

The online version of this article (10.1186/s40694-018-0060-7) contains supplementary material, which is available to authorized users.

## Background

Genome-scale metabolic models have been successfully used as tools for guiding metabolic engineering, analyzing cellular phenotypes and contextualizing omics data [1–3]. For all of these tasks, high quality reconstructions are needed to minimize problems introduced by errors in the model. Today, there are multiple approaches for model generation spanning from classic manual model building, semi-automated and fully-automated generation of genome-scale models [1, 4, 5]. The latter approaches are especially valuable for new or under-characterized species as the genome sequence can form the basis for the construction of a draft genome-scale model. For well-characterized species, the classic model building approach provides the opportunity of integrating available experimental knowledge into a structured framework, allowing for consistency checking and identification of knowledge gaps. The probably best curated genome-scale models are available for widely used model species such as *E. coli* and *S. cerevisiae*. The consensus reconstruction of *S. cerevisiae* has been curated in a community-driven effort for several years and is able to simulate gene deletions and growth performance with high accuracy [6]. In this paper we aimed at establishing a community consensus genome-scale model of *A. niger* that enables researchers to run constraint-based analyses like prediction of gene knockout phenotypes or maximum yields under different conditions. While particular areas of metabolism in Aspergilli have attracted significant attention [7], there are still big gaps [8] in our understanding to be addressed.

*Aspergillus niger* described in 1867 by Van Tiegham, sparked considerable interest due to the observation of citric acid overproduction in the beginning of the last century [9]. Besides being an industrial work-horse in citric acid production, *A. niger* and its close relatives are also widely used hosts for enzyme production [10, 11]. Owing to the commercial interest in *A. niger* and its metabolic flexibility with respect to utilizable substrates, there has been sustained research to elucidate the metabolism of this organism. The first genome-scale model of *A. niger* was published by one of the authors [12], which has been built on a former reconstruction of central carbon metabolism of *A. niger*[13]. The original model has been widely used for a variety of applications, e.g. for modeling acid production [14] and predicting protein yields [15]. However, these modeling efforts are based on the state of the art in 2008. During the last decade, substantial amounts of research have been conducted on *A. niger* metabolism as well as on the metabolism of closely related fungi, which can be used to greatly improve the predictions and metabolic network of *A. niger*. Additionally, the organization of biological data has changed tremendously in the last decade. Standards for structuring models (e.g. SBML), identifying chemical reactions (E.C. numbers and KEGG identifiers), referencing literature (e.g. DOIs, Pubmed IDs, and PMC IDs), and identification of chemical compounds (ChEBI’s and InChI’s) have been established and/or updated, enabling a much higher degree of cross-referencing of information and establishing interlinked data structures.

Recently, a different update of the original genome-scale model has been published by Lu et al. [16]. The authors used a partially overlapping updating strategy. First, they updated the annotations of metabolites and reactions and balanced all unbalanced reactions. The authors then used the information from four databases to add new reactions to the model and update existing gene-protein-reaction associations (GPRs). While also making use of the structured information provided by those databases, we aimed for a systematic storage of the primary data in the model thereby ensuring long-term evolution of the model. In this work, we present an updated genome-scale model of *A. niger* that has undergone major revisions with respect to the metabolic coverage as well as the quality of gene assignments, and is in compliance with state-of-the-art data standards. The end result is a gold-standard curated and validated genome-scale model, incorporating the information of 876 publications.

## Results

### Update methodology and statistics

The aim of the update was to improve the original genome-scale model of *A. niger* [12], both with respect to coverage as well as with respect to the annotations included in the model, in particular the assignment of genes to reactions. Furthermore, specific interests have been to include modeling of secreted secondary metabolites and the hundreds of proteins, which *A. niger* is known to produce. In a first step, metabolites have been annotated with their respective ChEBI identifier [17] to enable their unequivocal identification in relevant databases. The undissociated form of the individual metabolites has been used to avoid problems caused by the largely unknown proton stoichiometry of transport reactions. Reactions have been checked for mass balance accordingly and have been adapted where needed, in particular to include protons in all reactions where they are known to be present.

In a second step, the model has been updated based on primary literature, patents, as well as on information contained in the AspGD [18], BRENDA [19] and AMIGO2 [20] databases. The total number of publications in PubMed on *A. niger* has roughly doubled since the publication of the *i*MA871 model, and the abstracts of all these publications have been examined manually and complemented by additional searches. Manual searches for literature have been further complemented by comparing the genome sequence using the BLAST algorithm against the non-redundant patent sequence database [21] as well as the UniProtKB/Swiss-Prot database [22]. The experimental information included in those resources have been tracked back to the primary source and added in a structured way to the model with a reference to its origin.

**Fig. 1:**
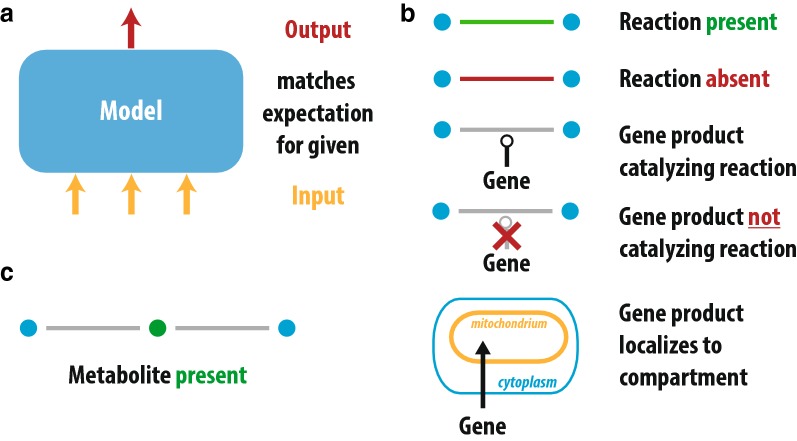
Types of experimental evidence included in the current version of the model. Panel A) depicts the concept of model test cases included in iJB1325. The test cases consist of a set of active input reactions, inactive genes as well as conditions for the test to pass. Inputs therefore refer to metabolites present in the growth medium or the knockout of a specific gene. Test conditions can be the ability to produce biomass or other compounds. Panel B) shows the different pieces of evidence stored in the model for genes and reactions while Panel C) depicts the information stored about metabolites

As an integrated part of the model update process, we have implemented the model in fully functional SBML. Genome-scale metabolic models are usually shared in the standardized SBML format [23]. This format is routinely read by popular modeling software packages thereby preventing the error prone process of custom model parsing [24]. While the standard focuses on the safe distribution of models, the XML basis of the format allows for the introduction of additional information without breaking the format. We have therefore further improved the association of literature and model reactions by integrating the references and their level of support directly in the SBML file. We separated the experimental data into two classes: Evidence items (see panels B and C in Fig. 1) and Test cases (see panel A in Fig. 1). Tests can be viewed as simple Input/Output tests and consist of a list of test conditions i.e. medium composition and gene knockouts and a list of reported outcomes that are tested for. With this setup, the differential growth of strains of *A. niger* on combinations of C- and N-sources can be saved in a structured way that can later be tested through simulation in an automated manner. Evidence items are used for the storage of information containing the presence/absence of a specific reaction or metabolite, as well as for the presence/absence of a connection between a gene and reaction. Additionally localization of a gene product to a specific compartment can be represented as well. Relevant information contained in the literature used for building the model has been saved as corresponding test case or evidence information.

As a third step, and in order to further improve the amount of available experimental information for *A. niger*, we employed phenotype arrays to screen the capability of *A. niger* spores to germinate and grow on 190 different C-sources as well as 95 N-sources using phenotype screening plates from Biolog Inc. If *A. niger* showed ability to grow on the substrate (Additional files 1 and 2), the model was updated to include the relevant catabolic pathway if possible. This led to a further addition of 34 pathways to the model. The absence of growth has not been used as information in the modelling process as this might be either caused by absence of transport, a missing catabolic route or lack of expression of the former two and might therefore represent a false negative result.

Fourthly, two other expanded models for *A. niger* have been published during the development of this one, one *de novo*-generated based on an advanced automated method [5], and one by Lu et al. [16], expanding our previous iMA871 model. We have analyzed the content of these models and integrated information from these where appropriate, thus generating a consensus-type model.

The update strategy described led to the expansion of the model with respect to several pathways (Table 1) as well as the update of pathways already included in the model. The final version presented here was named *A. niger* iJB1325. The comparison of the key statistics of the different models is shown in Table 2. The current update of the model includes 1325 genes and therefore adds 454 genes to the original model, which is on par with the model published by Lu et al. [16]. The number of metabolites has been increased by 773–1818 while including 1130 additional reactions. Overall experimental information from 876 sources has been included in the model, which represents an addition of 505 publications in comparison to our first model. The experimental information has been broken up into evidence items and test cases thereby allowing for the easy backtracking of the experimental information as well as for the automatic validation of the model structure, growth/production capabilities against the knowledge of the 876 publications used for building the model.

**Table 1:** Newly introduced pathways

Degradation	Biosynthesis	Secondary metabolites
Agmatine degradation	CoQ biosynthesis	Azanigerones
Amide degradation	Coprogen biosynthesis	Malformins
Aromatics degradation	Storage compounds	Nigragillin
Peroxisomal beta oxidation	Ferrichrome	Kotanin
Cyanide degradation	Iron assimilation	Funalenone
Galacturonic acid degradation	Lipoic acid biosynthesis	Pyranonigrin
Detoxification of compounds	Metabolite repair	Aurasperone
Glucuronate degradation	NAD biosynthesis	Tetraacetic acid lactone
Isoleucine degradation	Thiamin biosynthesis	
Leucine degradation	Vitamin metabolism	
Lipid degradation	Molybdenum cofactor	
Plant biomass degradation	Riboflavin biosynthesis	
Purine degradation		
Valine degradation		
L-Rhamnose metabolism		

### Strain-specific model implementations

In order to provide strain-specific models for the most commonly used strains of *A. niger* we generated individual models for the sequenced strains ATCC 1015, and CBS 513.88 (see Additional files 2 and 3). Reciprocal best blast hits have been used to transfer reaction assignments and the experimental evidence from the development model to the individual models.There are 18 genes in ATCC1015 that have no hit in the CBS513.88 genome (see Additional file 4: Table 4).

### Validation, iterative improvements, and test cases

In the iJB1325 model, we included 471 test cases that can be run when updating the model in order to ensure consistency with the information that has been used for building the model. Those tests mainly comprise absence or presence of growth on different combinations of carbon and nitrogen sources (392 cases). A smaller set consists of tests for gene deletion phenotype i.e. absence or presence of growth of deletion mutants (73 cases). An even smaller number of tests comprise overall system checks i.e. possibility to produce biomass precursors, no growth in the absence of known essential medium components, and a check for the possibility to oxidize fatty acids in the peroxisome (6 cases). Running all tests with the current version of the model leads to 373 (79%) passing and 98 (21%) failing test cases. The failing tests consist of 75 cases failing due to an unknown metabolic pathway, 15 failing due to inconsistencies between experiments, and 8 tests failing due to reasons we have not been able to determine (see Additional file 5 for more information).

**Table 2:** Table depicting key statistics of the different models

Model	iMA873	iJB1325	CoReCo	iHL1210
*Reactions*				
Total	1380	2320	4917	1764
Transport	189	447	0	285
Boundary	0	385	148	189
Unbalanced	40	68	148	–
Annotated	1013	1239	0	–
No genes	340	604	3049	–
Evidence for presence	–	767	–	–
Known gene	–	654	–	–
*Metabolites*				
Total	1084	1818	4025	1254
Annotated	0	1533	0	
Dead-End	270	295	1739	
*Genes*				
Total	871	1325	4533	1210
Verified location	–	296	–	–
Predicted location	–	107		
Known function	–	707	–	–
*Evidences*				
Total	–	3482	–	–
Gene-reaction	–	1677	–	–
Metabolite presence	–	333	–	–
Reaction presence	–	539	–	–
Gene-compartment	–	907	–	–
*Test cases*				
Total	–	471	–	99
Passing	–	373	–	83
Failing	–	98	–	16
*References*				
Total	371	876	–	–

Having a collection of test conditions enables the identification of missing reactions in the model. One such example is the utilization of L-histidine as single N-source in combination with D-galactose. This was reported by Hayer et al. [25] as well as in combination with glycerol by Steinberg [26]. Growth on L-histidine in combination with glucose can also be observed in our own phenotype screening arrays. The previous version of the model was incapable of simulating growth on L-histidine as single N-source. The metabolic reactions involved in the utilization of L-histidine have not been reported to our knowledge. In *A. nidulans*, the presence of a histidase (histidine ammonia-lyase, E.C. 4.3.1.3) has been reported, but the corresponding gene remains to be identified. Using the reviewed entries in Uniprot, we could identify three candidate genes for histidase in *A. niger* (JGI *A. niger* ATCC 1015 (Aspni7) ProteinIDs 1129557, 1126350 and 1081533). Sequence comparison with the characterized enzyme from *P. putida* [27] showed high conservation for the active site residues for the three candidates (see Additional file 6); therefore, all three candidate genes have been added to the model.

Another example for the identification of missing reactions in the model is the growth on L-methionine or L-cysteine as single N-source. Growth of *A. niger* on both nitrogen sources has been observed by Hayer et al. 2014 [25] as well as in our Biolog experiments. Failing tests for growth of *A. niger* on those two N-sources hinted to the absence of the corresponding pathway. Evidence shown in *A. nidulans* by Sienko et al. [28] hints towards the degradation of L-methionine towards L-cysteine by a reverse transsulfuration reaction involving the genes *mecA* and *mecB*. Putative orthologs for these genes are present in *A. niger*. The metabolic fate of L-cysteine as single N-source has to our knowledge not been demonstrated conclusively in *A. niger* or any related fungus. We therefore did not include a L-cysteine degradation pathway and left this gap for future improvements.

Another example of a failing test is the no growth phenotype for the $\Delta$*gaaA* strain on  D-galacturonate. Besides the *gaaA* gene, the putative *H. jecorina* ortholog (JGI *A. niger* ATCC 1015 (Aspni7) transcriptID 1109007) has been included due to sequence similarity as a  D-galacturonic acid reductase. The experimental data reported by Mojzita et al. [29] however demonstrate that a strain with a defect in *gaaA* is not able to grow on  D-galacturonate, indicating that the alternative gene either is not a  D-galacturonate reductase, or is not expressed under the experimental condition. However, Alazi et al. [30] found only a reduction of growth in the $\Delta$*gaaA* strain indicating partial redundancy of the pathway. The putative ortholog has therefore been kept in the model.

### Evidence-based support for reactions

In the iJB1325 model, we included several levels of evidence from the literature to enable to continuous testing of model connectivity during future improvements. The different types of evidence included in the model are depicted in Fig. 1. For a format description of the evidence items, see Additional file 7. For the assignment of genes to individual metabolic reactions, primary literature for *A. niger* as well as related species has been used. Making use of the evidence code ontology [31], we included a measure of certainty for the individual connections. The localization of the individual proteins has been predicted using Mitofates [32] for mitochondrial proteins as well as PTS1 predictor [33] for peroxisomal localization predictions. In order to identify secreted proteins, we compiled a list of published proteomics experiments on *A. niger* [34–44]. Extracellular localization has been considered a true positive if the presence in the extracellular space has been reported by three independent publications. The prediction of localizations is complicated by the fact that some proteins are localized in multiple compartments by alternative translational start sites [45], differential splicing [46, 47] as well as stop-codon readthrough [48]. However due to the lack of experimental validation of the localization of most proteins, predictions for mitochondrial and peroxisomal localization have been included as best guess.

Additionally, evidence items for the presence of individual metabolites in *A. niger* have been included where reported in the literature. The presence and absence of reactions have also been included when reported. In total, we have been able to include 3482 pieces of evidence that link individual components inside the model and are associated with an evidence code as a measure of certainty, a small description where appropriate describing the underlying experiment, and a link to the resource the conclusion has been drawn from. This small summary allows for a quick assessment of the quality of the gene assignment and thereby easing the interpretation.

**Fig. 2:**
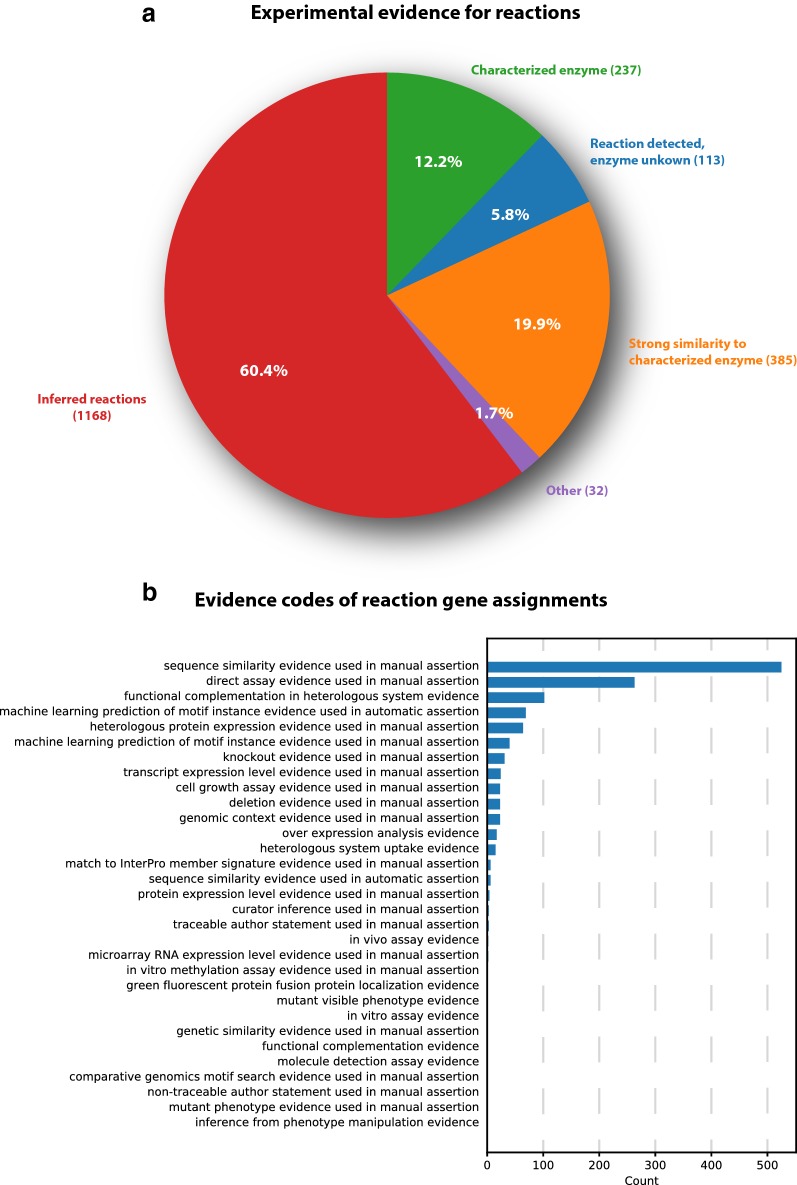
Experimental support for individual reactions. **a** Depicts the strongest experimental support for the presence of individual reactions in the model. The categories according to decreasing experimental support are: “Characterized enzyme”, “Measured, but unknown enzyme”, “Strong similarity to characterized enzyme”, “Other” and “No experimental evidence”. **b** Shows the evidence codes associated with the individual reaction gene assignments

**Fig. 3:**
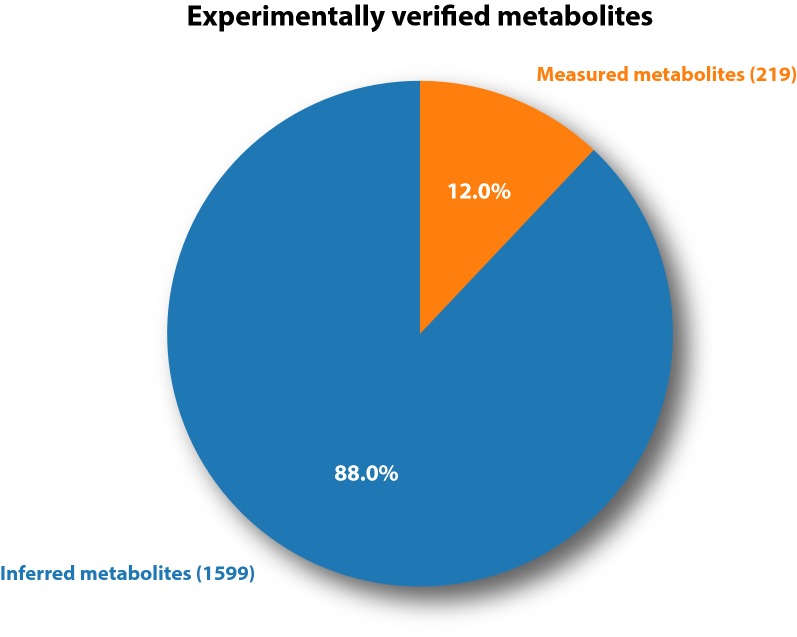
Experimental support for individual metabolites

The distribution of the experimental support of the individual reactions is shown in Fig. 2 panel A. About 19% of the reactions included in the model have direct experimental support by either being measured *in vitro* or having a gene with a confirmed activity assigned. Another 17% of the reactions have a gene assigned based on a strong similarity to a characterized enzyme in a closely related species. These gene reactions associations are mainly derived from characterized genes of the related fungal model organisms *A. nidulans* and *A. fumigatus*. The remaining reactions either have no genes assigned or are inherited from the iMA871 model as the best candidates for a given reaction based on sequence similarity to characterized enzymes or domain predictions. The distribution of the individual evidence codes of all reaction-gene-assignments is depicted in Fig. 2 panel B. The experimental support for individual metabolites are depicted in Fig. 3. About 12% of the metabolites included in the model have been measured experimentally leaving 88% of the metabolites that were inferred during the reconstruction process.

### Application of the model network for transcriptomics data analysis

In addition to our 471 modeling test cases, we wanted to acknowledge that a significant application of genome-scale models is the use of the underlying metabolic networks for data analysis and interpretation [2, 49]. Using the current version of our genome-scale model, we analyzed an compendium of transcriptomics data based on published studies using a microarray developed for the *A. niger* ATCC 1015 strain [14, 15, 50–53], as well as a compendium based on an *A. niger* CBS 513.88 array data [54].

**Fig. 4:**
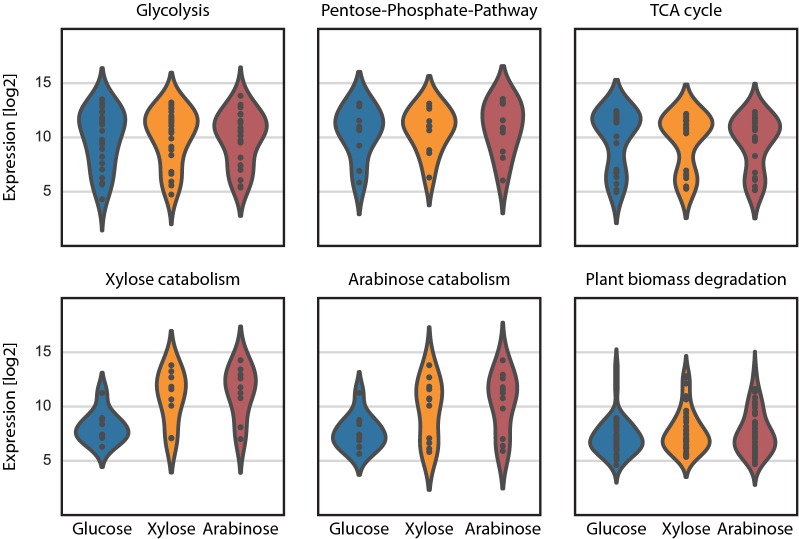
Change in transcription level of the genes assigned to metabolic pathways under different conditions. Violin plots showing the change of expression of genes involved in different metabolic pathways depending on the carbon source used

One possible application for a genome-scale model is the analysis of expression data using the gene-protein-reaction (GPR) associations included in the model. The grouping of reactions into pathways, thereby also grouping the associated genes, allows for overall contextualization of expression changes in transcriptomics data as depicted in Fig. 4 for the ATCC 1015 transcriptome data (see Additional file 8: Figure 8 for the corresponding analysis for the CBS 513.88 dataset). In both cases, plotting the expression level of different subnetworks demonstrates the expected up-regulation of the genes associated with  D-xylose and L-arabinose catabolism on those two carbon sources while there appears to be no overall change of genes involved in glycolysis or the TCA cycle. Some of the genes associated with plant biomass degradation which consists mainly of polysaccharide degrading enzymes are upregulated, but the majority of these genes does not show a change in expression under these conditions. Using the correlation of gene expression levels to individual pathways the transporter with the JGI Aspni7 TranscriptID 1178899 appeared to be co-regulated with the known members of the *ada* cluster that has been characterized by Li et al. [55]. The transporter is located next to the *adaA* gene and therefore we named this gene *adaE* and included it as a putative TAN-1612 transporter in the model.

Transcriptomics data can also be used for the validation of genome-scale models as performed by Lu et al. [16]. Unfortunately only the part of the data set covering the genes in their model has been published, therefore not allowing a direct comparison of the model presented here to their transcriptomics data set.

## Discussion

*Aspergillus niger* has been used as a biotechnological workhorse for about a century producing citric acid and several enzymes in high amounts. During this time much research has been dedicated to shedding light on the underlying metabolic network. The resulting information has been published in several hundreds to thousands of papers containing the individual pieces of the puzzle. With the advent of the genomic era, databases have been developed that try to catalog the literature information on the individual genes [18]. However, in order to be able to analyze and understand the metabolism at a systems level, integration into a coherent framework is needed. Genome-scale models can provide such a framework in which the knowledge about metabolism can be integrated, tested for logical consistency, and predictions made.

In this study we present an update of the genome-scale model that has been developed in our group almost a decade ago [12]. The updated model integrates the knowledge from 876 publications into a consistent framework, thereby representing the experimentally best supported model currently available. As the model update process represents an iterative process spanning years to decades, a sustainable way of keeping track of the information used in the curation process is needed. With this in mind, we created a new structure to store all information used for the model building in the model file, thereby allowing users to modify the model and check the changed version against all literature information in an automated fashion. This approach led to the inclusion of 3482 evidence items and 471 test cases. Whereas test cases allow for the representation of both quantitative and qualitative macroscopic observations representing overall model predictions (e.g. growth or production capacities), evidence items represent the experimental support for the model structure (e.g. presence/absence of a compound or biochemical reaction). In order to allow backtracking, we stored the primary literature references with the individual evidence items and test cases. As the information used for model building is currently not shared in a standardized form, we aimed at extending the SBML format [23] in a manner compliant with the specification, making it usable by other researchers. To our knowledge this is the first time a model has been presented, which allows for efficient continuous improvement making use of automated testing. While this strategy increases the complexity of the reconstruction, it allows backtracking of the experimental information used for the model building. This feature makes the model a true knowledge-base for *A. niger* metabolism that is not only valuable for future improvements, but also provides a structured way for the search of existing experimental knowledge. The usefulness of having this information has been demonstrated by the ability for identifying missing reactions, as well as in checking the connectivity of the network.

The model development has been focused on the update of the information and gene assignments for the *A. niger* ATCC1015 strain. However, as the strains are very similar and in order to allow the utilization of the model by more users, we used reciprocal best blast hits to translate the genes in the model to the identifier of the *A. niger* CBS513.88 strain.

Recently a model has been published by Lu et al. [16] that already included improvements to the metabolite annotation and reaction quality that are presented in the current publication. The authors also validated their model using 99 growth tests for different carbon and nitrogen sources where the model performed successfully in 83 of 99 tests. We also included tests for those growth conditions if not already present in the model. While 373 passing tests out of 471 seems worse than the relation presented by Lu et al. the number of failing tests is explained by the wider coverage thereby including substrates with unknown metabolic pathways that could not be included in the model. Inclusion of these test cases leads to presence of a substantial number of dead-end metabolites in the current version of the model which are frequently removed from genome-scale models as reactions associated with those metabolites are guaranteed to carry no flux. We decided to keep those metabolites in the model as points for future model improvement and to contain the experimental data that led to the inclusion of those reactions.

One important aspect of eukaryotic metabolism is the compartmentalization of reactions into different organelles. Unfortunately, information about the subcellular localization of individual enzymes is only scarcely available in *Aspergillus niger*. Another existing challenge for genome-scale modelling in filamentous fungi is the presence of multiple seemingly orthologous genes for many metabolic functions. The existence of these multiple copies poses the challenge to assign the individual contribution of those genes to the metabolic activity. One way of identifying the best candidate for a function is comparing the expression values of a gene to verified genes upstream or downstream in the same pathway. If known constituents of the pathway are expressed at a very high level (e.g. see Glycolysis or TCA cycle in Fig. 4) missing members are expected to have an expression level in the same range as the known members. We tried using transcriptomics data in such a manner as proxy for the individual contribution. This proved to be challenging as for most reactions the expected activity level is unknown, as well as for many genes the correct assignment is not evident. Due to the mentioned difficulties we did not include the transcript level as experimental evidence in the majority of the reactions. With the development of the CRISPR/Cas9 system in filamentous fungi, large-scale genetic manipulations become increasingly feasible thereby allowing the assessment of the contribution of individual genes to a specific phenotype on a genome-scale model. The development of a large-scale knockout library would be an interesting project for the validation of the genome-scale model presented here.

## Conclusion

Here we presented the largest and most thoroughly curated genome-scale model of *A. niger* metabolism to date. The model has been built on an extensive body of primary literature which has been structured and saved along with the model. We therefore extended the SBML format of the model to include the literature information about reactions and gene functions resulting in 1677 evidence items for gene reaction links, 539 items for the presence or absence of reactions, and 907 items for subcellular protein localization. At the same time we integrated the growth capabilities of *A. niger* as reported in the literature with our own experimental data, leading to the validation of the model against 471 test cases. From this validated model, strain specific models have been generated for *A. niger* ATCC1015, and CBS513.88. Finally, the model has been demonstrated to be useful for the interpretation of -omics data providing the metabolic context of the individual genes .

## Methods

### Software

During the process of updating the model we used a newly developed software for the editing of the model. This software will be published separately and is based on existing open source software packages COBRApy [56] and Escher [57] for simulation and visualization, respectively. The software is already available at https://github.com/JuBra/GEMEditor. Users interested in testing, updating and viewing the experimental information included in the model are referred to the GEMEditor wiki for instructions about installation and analysis of the model.

### Bibliomic data

In order to identify extracellular proteins, available extracellular proteomics data has been collected [34–44]. Characterized proteins have been identified from primary literature, individual patents by searching for patents on *Aspergillus niger* as well as by blasting against the non-redundant patent sequence database [21]. Proteins not accounted for in the model have additionally been blasted against the UniProtKB/Swiss-Prot database [22] to identify proteins with known functions.

### Integration of public experimental data

Experimental data have been gathered from several literature resources. We combined all available proteomics information for *A. niger* for assessing the sub-cellular localization of different gene products. Additionally, subcellular prediction of proteins has been performed using MitoFates [32] for mitochondrial and PTS1 predictor [33] for peroxisomal localization prediction. The analysis of the transcriptomics data has been performed on a dataset for *A. niger* ATCC1015 collected from [14, 15, 50–53] and for *A. niger* CBS513.88 (GEO accession number GSE98572, Samples: GSM2600962, GSM2600963, GSM2600941, GSM2600942, GSM2600992 and GSM2600993) collected from Gruben et al. [58].

### Phenotype arrays

Screening for growth on nitrogen and carbon-sources has been performed using the phenotype plates PM1, PM2A and PM3B from Biolog Inc. The plates have been prepared according to the manufacturers manual with the spore density being adjusted to $10^7$ spores per ml. The plates have been incubated at $28\,^{\circ }\hbox {C}$ for up to 10 days. Growth has been assessed by inspecting the plates visually for sporulation, see Additional files 1 and 9.

### Test cases

The test cases introduced in the current version of the model consist of a list of settings representing the simulation conditions, a list of deactivated genes and a list of outcomes the resulting solution is checked against. All boundary reactions that are not specified in the settings list are set to only being able to consume the metabolite. Outcomes are a combination of a specific reaction, a greater than or less than modifier and a numerical value (see Additional file 10).

### Model simulation

Test simulations have been run using the FBA or pFBA method as implemented in COBRApy [56] (see Additional file 11).

## Additional file

**Additional file 1.** Image of Biolog PM3B screening plate. The image shows a picture of a Biolog PM3B screening plate inoculated with* A. niger* ATCC1015 after 4 days of incubation at 32 °C. Nitrogen sources on plate: Control; Ammonia; Nitrite; Nitrate; Urea; Biuret; L-Alanine;  L-Arginine;  L-Asparagine;  L-Aspartic Acid;  L-Cysteine;  L-Glutamic Acid;  L-Glutamine; Glycine;  L-Histidine;  L-Isoleucine;  L-Leucine;  L-Lysine;  L-Methionine;  L-Phenylalanine;  L-Proline;  L-Serine;  L-Threonine;  L-Tryptophan;  L-Tyrosine;  L-Valine; D-Alanine;  D-Asparagine;  D-Aspartic Acid;  D-Glutamic Acid;  D-Lysine;  D-Serine;  D-Valine;  L-Citrulline;  L-Homoserine;  L-Ornithine; N-Acetyl- L-Glutamic Acid; N-Phthaloyl- L-Glutamic Acid;  L-Pyroglutamic Acid; Hydroxylamine; Methylamine; N-Amylamine; N-Butylamine; Ethylamine; Ethanolamine; Ethylenediamine; Putrescine; Agmatine; Histamine; beta-Phenylethyl- amine; Tyramine; Acetamide; Formamide; Glucuronamide; D, L-Lactamide;  D-Glucosamine;  D-Galactosamine;  D-Mannosamine; N-Acetyl- D-Glucosamine; N-Acetyl- D-Galactosamine; N-Acetyl- D-Mannosamine; Adenine; Adenosine; Cytidine; Cytosine; Guanine; Guanosine; Thymine; Thymidine; Uracil; Uridine; Inosine; Xanthine; Xanthosine; Uric Acid; Alloxan; Allantoin; Parabanic Acid; D, L-alpha-Amino-N-Butyric Acid; gamma-Amino-N-Butyric Acid; epsilon-Amino-N-Caproic Acid; D, L-alpha-Amino- Caprylic Acid; delta-Amino-N-Valeric Acid; alpha-Amino-N-Valeric Acid; Ala-Asp; Ala-Gln; Ala-Glu; Ala-Gly; Ala-His; Ala-Leu; Ala-Thr; Gly-Asn; Gly-Gln; Gly-Glu; Gly-Met; Met-Ala.

**Additional file 2.** Genome-scale model for Aspergillus niger ATCC1015. Model file in SBML level 3 version 1.

**Additional file 3.** Genome-scale model for Aspergillus niger CBS513.88. Model file in SBML level 3 version 1.

**Additional file 4.** Gene differences between strain ATCC1015 and CBS513.88. The list shows the genes for which no reciprocal best blast hit could be identified in* A. niger* CBS513.88.

**Additional file 5.** Failing test cases. The file consists of the currently failing test cases of the model with an explanation why they are failing.

**Additional file 6.** Sequence comparison of the candidates for histidase. Multiple sequence alignment of the candidates for the histidase activity.

**Additional file 7.** Example of the format for storing experimental data. Example entries for the experimental data as saved in the current version of the model.

**Additional file 8.** Changes in expression of genes belonging to different pathways. The change of gene expression in the CBS513.88 model.

**Additional file 9.** Image of Biolog PM1 screening plate. The image shows a picture of a Biolog PM1 screening plate inoculated with* A. niger* ATCC1015 after 9 days of incubation at 32 °C. Carbon sources on plate: Control; L-Arabinose; N-Acetyl-D-Glucosamine; D-Saccharic Acid; Succinic Acid; D-Galactose;  L-Aspartic Acid;  L-Proline; D-Alanine; D-Trehalose; D-Mannose; Dulcitol; D-Serine; D-Sorbitol; Glycerol;  L-Fucose; D-Glucuronic Acid; D-Gluconic Acid; D, L-alpha-Glycerol- Phosphate; D-Xylose;  L-Lactic Acid; Formic Acid; D-Mannitol;  L-Glutamic Acid; D-Glucose-6-Phosphate; D-Galactonic Acid-gamma-Lactone; D, L-Malic Acid; D-Ribose; Tween 20;  L-Rhamnose; D-Fructose; Acetic Acid; alpha-D-Glucose; Maltose; D-Melibiose; Thymidine;  L-Asparagine; D-Aspartic Acid; D-Glucosaminic Acid; 1,2-Propanediol; Tween 40; alpha-Keto-Glutaric Acid; alpha-Keto-Butyric Acid; alpha-Methyl-D-Galactoside; alpha-D-Lactose; Lactulose; Sucrose; Uridine;  L-Glutamine; m-Tartaric Acid; D-Glucose-1-Phosphate; D-Fructose-6-Phosphate; Tween 80; alpha-Hydroxy Glutaric Acid-gamma-Lactone; alpha-Hydroxy Butyric Acid; beta-Methyl-D-Glucoside; Adonitol; Maltotriose; 2-Deoxy Adenosine; Adenosine; Glycyl- L-Aspartic Acid; Citric Acid; m-Inositol; D-Threonine; Fumaric Acid; Bromo Succinic Acid; Propionic Acid; Mucic Acid; Glycolic Acid; Glyoxylic Acid; D-Cellobiose; Inosine; Glycyl- L-Glutamic Acid; Tricarballylic Acid;  L-Serine;  L-Threonine;  L-Alanine;  L-Alanyl-Glycine; Acetoacetic Acid; N-Acetyl-beta-D-Mannosamine; Mono Methyl Succinate; Methyl Pyruvate; D-Malic Acid;  L-Malic Acid; Glycyl- L-Proline; p-Hydroxy Phenyl Acetic Acid; m-Hydroxy Phenyl Acetic Acid; Tyramine; D-Psicose;  L-Lyxose; Glucuronamide; Pyruvic Acid;  L-Galactonic Acid-gamma-Lactone; D-Galacturonic Acid; Phenylethyl-amine; 2-Aminoethanol.

**Additional file 10.** Correlation of members of the Anthracenone cluster. The file shows the expression values for individual genes that have been identified to be part of the ada cluster. The adaD gene has not been included in the source microarray of the data.

**Additional file 11.** Export of the evidence items contained in the ATCC1015 model. Gene IDs correspond to ATCC1015 Aspni7 transcriptIds.
