# Supplementary figures and images for: A community-driven reconstruction of the Aspergillus niger metabolic network
Source: Fungal Biol Biotechnol. 2018 Sep 26;5:16. doi: 10.1186/s40694-018-0060-7 (PMC6158834; doi:10.1186/s40694-018-0060-7)

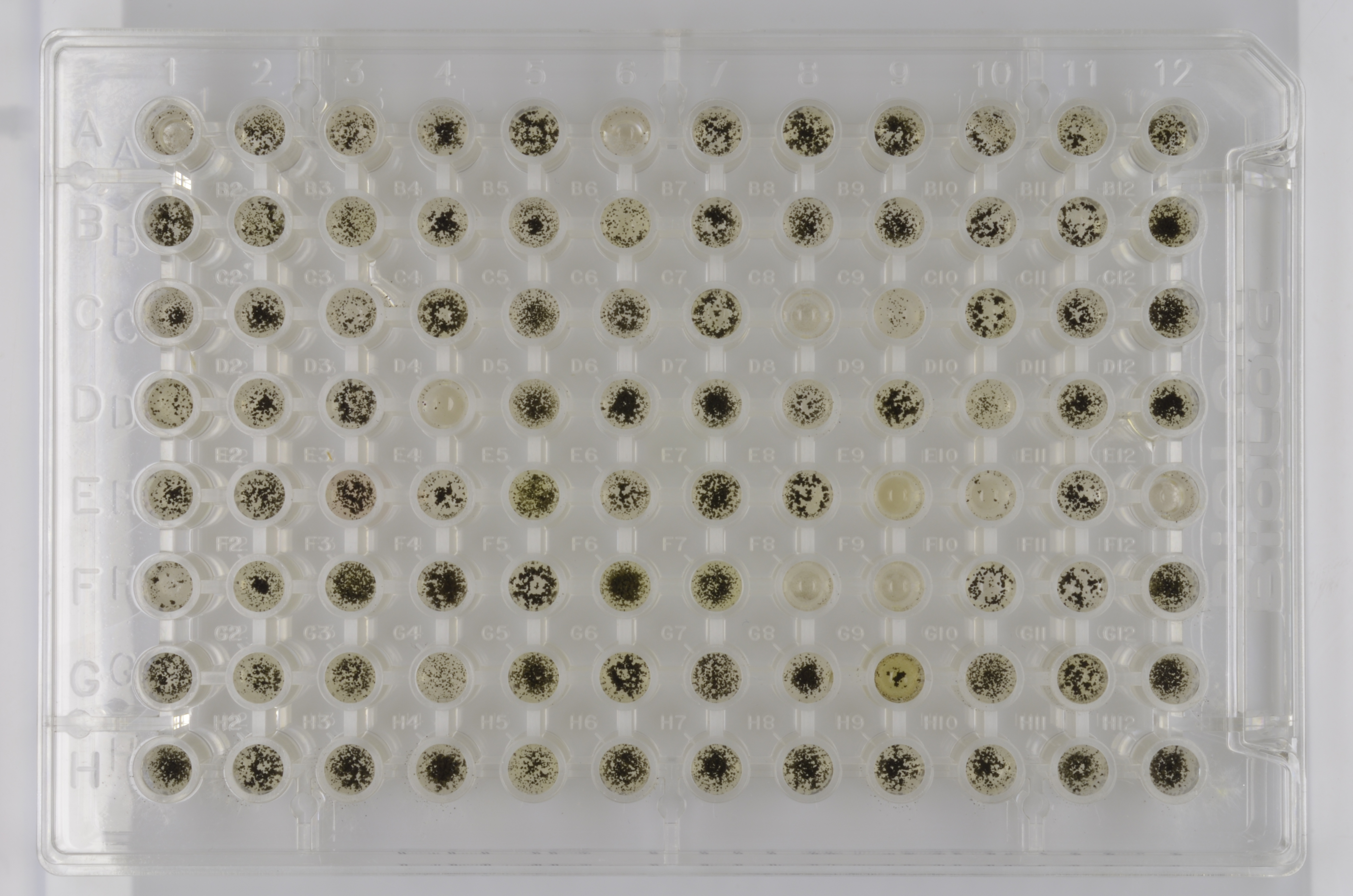

Supplement: Supplementary file 1 — Additional file 1. Image of Biolog PM3B screening plate. The image shows a picture of a Biolog PM3B screening plate inoculated with A. niger ATCC1015 after 4 days of incubation at 32 °C. Nitrogen sources on plate: Control; Ammonia; Nitrite; Nitrate; Urea; Biuret; L-Alanine; L-Arginine; L-Asparagine; L-Aspartic Acid; L-Cysteine; L-Glutamic Acid; L-Glutamine; Glycine; L-Histidine; L-Isoleucine; L-Leucine; L-Lysine; L-Methionine; L-Phenylalanine; L-Proline; L-Serine; L-Threonine; L-Tryptophan; L-Tyrosine; L-Valine; D-Alanine; D-Asparagine; D-Aspartic Acid; D-Glutamic Acid; D-Lysine; D-Serine; D-Valine; L-Citrulline; L-Homoserine; L-Ornithine; N-Acetyl- L-Glutamic Acid; N-Phthaloyl- L-Glutamic Acid; L-Pyroglutamic Acid; Hydroxylamine; Methylamine; N-Amylamine; N-Butylamine; Ethylamine; Ethanolamine; Ethylenediamine; Putrescine; Agmatine; Histamine; beta-Phenylethyl- amine; Tyramine; Acetamide; Formamide; Glucuronamide; D, L-Lactamide; D-Glucosamine; D-Galactosamine; D-Mannosamine; N-Acetyl- D-Glucosamine; N-Acetyl- D-Galactosamine; N-Acetyl- D-Mannosamine; Adenine; Adenosine; Cytidine; Cytosine; Guanine; Guanosine; Thymine; Thymidine; Uracil; Uridine; Inosine; Xanthine; Xanthosine; Uric Acid; Alloxan; Allantoin; Parabanic Acid; D, L-alpha-Amino-N-Butyric Acid; gamma-Amino-N-Butyric Acid; epsilon-Amino-N-Caproic Acid; D, L-alpha-Amino- Caprylic Acid; delta-Amino-N-Valeric Acid; alpha-Amino-N-Valeric Acid; Ala-Asp; Ala-Gln; Ala-Glu; Ala-Gly; Ala-His; Ala-Leu; Ala-Thr; Gly-Asn; Gly-Gln; Gly-Glu; Gly-Met; Met-Ala. [file 40694_2018_60_MOESM1_ESM.jpg]

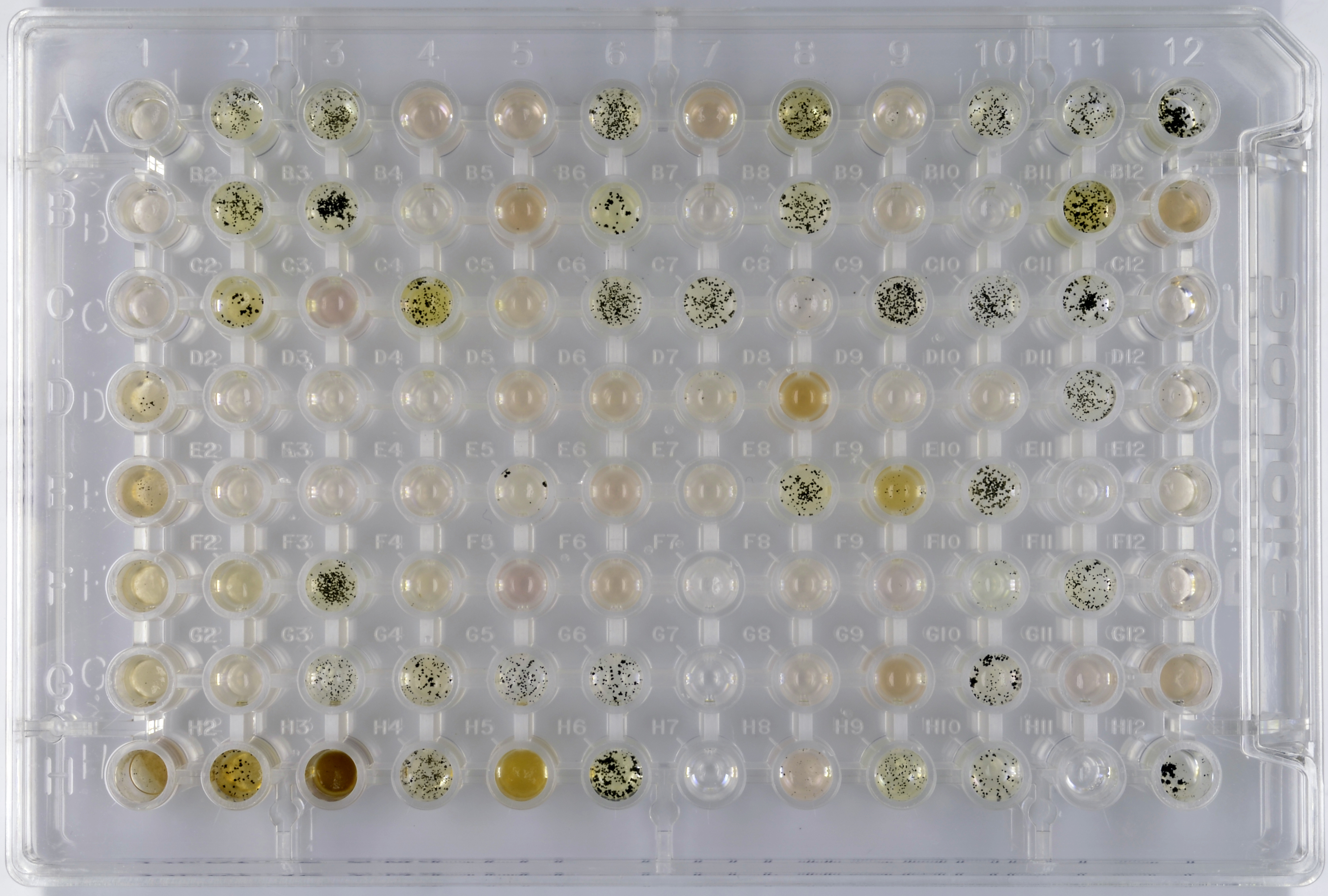

Supplement: Supplementary file 9 — Additional file 9. Image of Biolog PM1 screening plate. The image shows a picture of a Biolog PM1 screening plate inoculated with A. niger ATCC1015 after 9 days of incubation at 32 °C. Carbon sources on plate: Control; L-Arabinose; N-Acetyl-D-Glucosamine; D-Saccharic Acid; Succinic Acid; D-Galactose; L-Aspartic Acid; L-Proline; D-Alanine; D-Trehalose; D-Mannose; Dulcitol; D-Serine; D-Sorbitol; Glycerol; L-Fucose; D-Glucuronic Acid; D-Gluconic Acid; D, L-alpha-Glycerol- Phosphate; D-Xylose; L-Lactic Acid; Formic Acid; D-Mannitol; L-Glutamic Acid; D-Glucose-6-Phosphate; D-Galactonic Acid-gamma-Lactone; D, L-Malic Acid; D-Ribose; Tween 20; L-Rhamnose; D-Fructose; Acetic Acid; alpha-D-Glucose; Maltose; D-Melibiose; Thymidine; L-Asparagine; D-Aspartic Acid; D-Glucosaminic Acid; 1,2-Propanediol; Tween 40; alpha-Keto-Glutaric Acid; alpha-Keto-Butyric Acid; alpha-Methyl-D-Galactoside; alpha-D-Lactose; Lactulose; Sucrose; Uridine; L-Glutamine; m-Tartaric Acid; D-Glucose-1-Phosphate; D-Fructose-6-Phosphate; Tween 80; alpha-Hydroxy Glutaric Acid-gamma-Lactone; alpha-Hydroxy Butyric Acid; beta-Methyl-D-Glucoside; Adonitol; Maltotriose; 2-Deoxy Adenosine; Adenosine; Glycyl- L-Aspartic Acid; Citric Acid; m-Inositol; D-Threonine; Fumaric Acid; Bromo Succinic Acid; Propionic Acid; Mucic Acid; Glycolic Acid; Glyoxylic Acid; D-Cellobiose; Inosine; Glycyl- L-Glutamic Acid; Tricarballylic Acid; L-Serine; L-Threonine; L-Alanine; L-Alanyl-Glycine; Acetoacetic Acid; N-Acetyl-beta-D-Mannosamine; Mono Methyl Succinate; Methyl Pyruvate; D-Malic Acid; L-Malic Acid; Glycyl- L-Proline; p-Hydroxy Phenyl Acetic Acid; m-Hydroxy Phenyl Acetic Acid; Tyramine; D-Psicose; L-Lyxose; Glucuronamide; Pyruvic Acid; L-Galactonic Acid-gamma-Lactone; D-Galacturonic Acid; Phenylethyl-amine; 2-Aminoethanol. [file 40694_2018_60_MOESM9_ESM.jpg]

Expression profile of the ada cluster

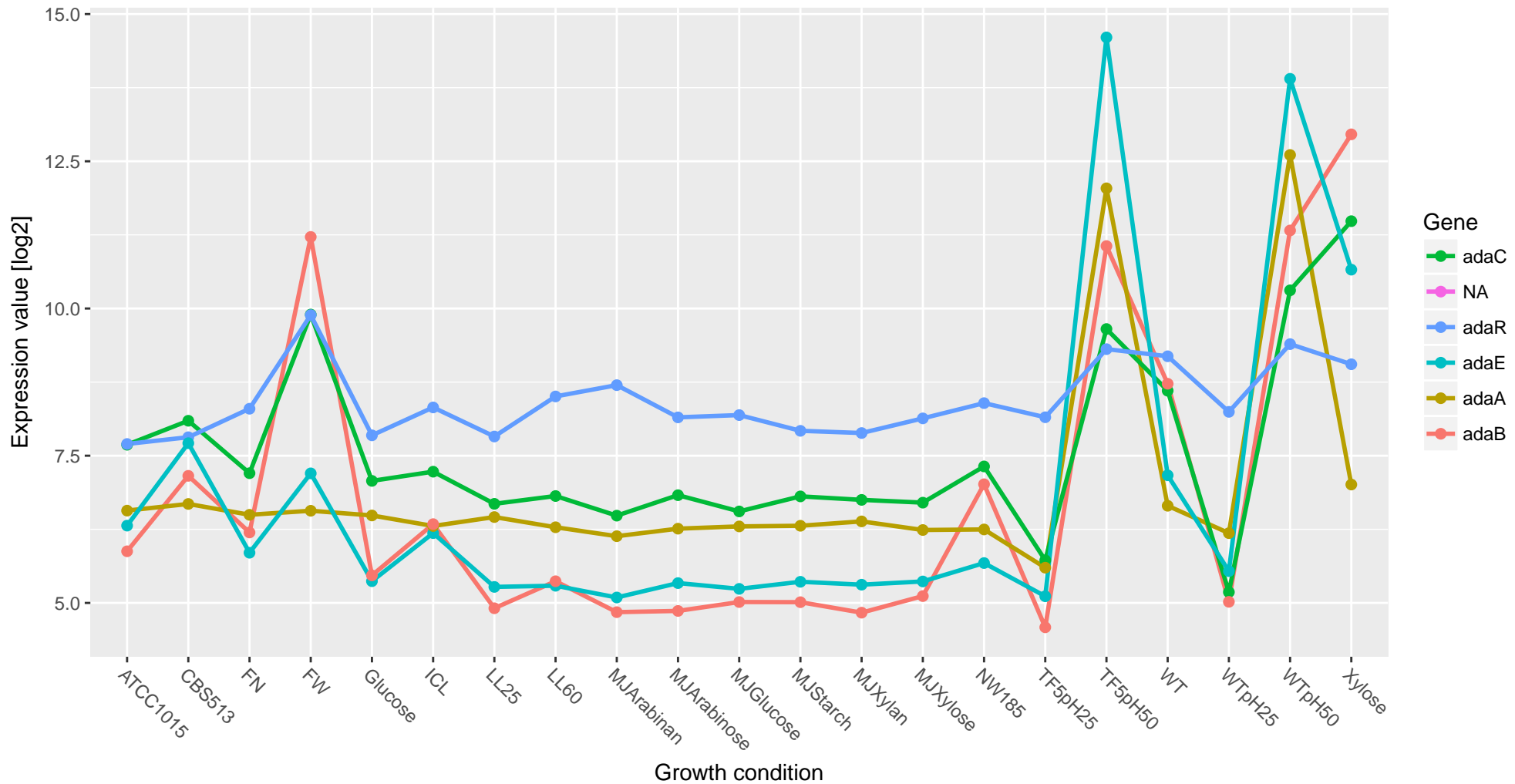

Supplement: Supplementary file 10 — Additional file 10. Correlation of members of the Anthracenone cluster. The file shows the expression values for individual genes that have been identified to be part of the ada cluster. The adaD gene has not been included in the source microarray of the data. [file 40694_2018_60_MOESM10_ESM.pdf]
