# Supplementary material for: A community-driven reconstruction of the Aspergillus niger metabolic network
Source: Fungal Biol Biotechnol. 2018 Sep 26;5:16. doi: 10.1186/s40694-018-0060-7 (PMC6158834; doi:10.1186/s40694-018-0060-7)
Supplement: Supplementary file 6 — Additional file 6. Sequence comparison of the candidates for histidase. Multiple sequence alignment of the candidates for the histidase activity. [file 40694_2018_60_MOESM6_ESM.pdf]

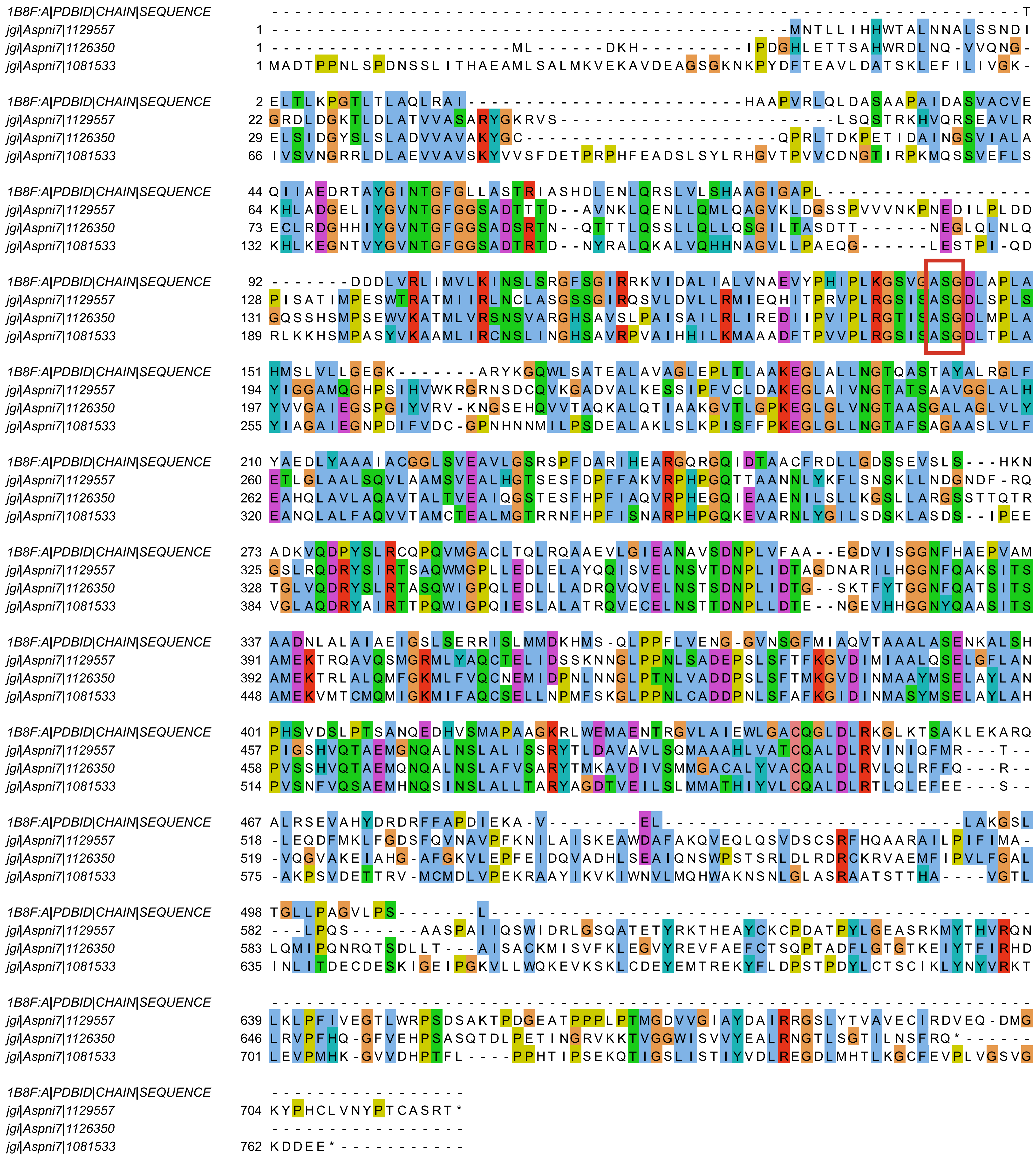
1B8F:A|PDBID|CHAIN|SEQUENCE ---T
jgi|Aspni7|1129557 1 ---MNTLLIHHWTALNNALSSNDI
jgi|Aspni7|1126350 1 ---------------------ML------DKH---------IPDGHLETTSAHWRDLNQ-VVQNG-
jgi|Aspni7|1081533 1 MADTPPNLSPDNSSLITHAEAMLSALMKVEKAVDEAGSGKNKPYDFTEAVLDATSKLEFILIVGK-
1B8F:A|PDBID|CHAIN|SEQUENCE 2 ELTLKPGTLTLAQLRAI------------------------HAAPVRLQLDASAAPAIDASVACVE
jgi|Aspni7|1129557 22 GRDLDGKTLDLATVVASARYGKRVS------------------------LSQSTRKHVQRSEAVLR
jgi|Aspni7|1126350 29 ELSIDGYSLSLADVVAVAKYGC----------------------QPRLTDKPETIDAINGSVIALA
jgi|Aspni7|1081533 66 IVSVNGRRLDLAEVVAVSKYVVSFDETPRPHFEADSLSYLRHGVTPVVCDNGTIRPKMQSSVEFLS
1B8F:A|PDBID|CHAIN|SEQUENCE 44 QIIAEDRTAYGINTGFGLLASTRIASHDLENLQRSLVLSHAAGIGAPL------------------
jgi|Aspni7|1129557 64 KHLADGELIYGVNTGFGGSADTTTD--AVNKLQENLLQMLQAGVKLDGSSPVVVNKPNEDILPLDD
jgi|Aspni7|1126350 73 ECLRDGHHIYGVNTGFGGSADSRTN--QTTTLQSSLLQLLQSGILTASDTT------NEGLQLNLQ
jgi|Aspni7|1081533 132 KHLKEGNTVYGVNTGFGGSADTRTD--NYRALQKALVQHHNAGVLLPAEQG------LESTPI-QD
1B8F:A|PDBID|CHAIN|SEQUENCE 92 -------DDDLVRLIMVLKINSLSRGFSGIRRKVIDALIALVNAEVYPHIPLKGSVGASGDLAPLA
jgi|Aspni7|1129557 128 PISATIMPESWTRATMIIRLNCLASGSSGIRQSVLDVLLRMIEQHITPRVPLRGSISASGDLSPLS
jgi|Aspni7|1126350 131 GQSSHSMPSEWVKATMLVRSNSVARGHSAVSLPAISAILRLIREDIIPVIPLRGTISASGDLMPLA
jgi|Aspni7|1081533 189 RLKKHSMPASYVKAAMLIRCNSLINGHSAVRPVAIHHILKMAAADFTPVVPLRGSISASGDLTPLA
1B8F:A|PDBID|CHAIN|SEQUENCE 151 HMSLVLLGEGK-------ARYKGQWLSATEALAVAGLEPLTLAAKEGLALLNGTQASTAYALRGLF
jgi|Aspni7|1129557 194 YIGGAMQGHPSIHVWKRGRNSDCQVKGADVALKESSIPFVCLDAKEGLAIVNGTATSAAVGGLALH
jgi|Aspni7|1126350 197 YVVGAIEGSPGIYVRV-KNGSEHQVVTAQKALQTIAAKGVTLGPKEGLGLVNGTAASGALAGLVLY
jgi|Aspni7|1081533 255 YIAGAIEGNPDIFVDC-GPNHNNMILPSDEALAKLSLKPISFFPKEGLGLLNGTAFSAGAASLVLF
1B8F:A|PDBID|CHAIN|SEQUENCE 210 YAEDLYAAAIACGGLSVEAVLGSRSPFDARIHEARGQRGQIDTAACFRDLLGDSSEVSLS---HKN
jgi|Aspni7|1129557 260 ETLGLAALSQVLAAMSVEALHGTSESFDPFFAKVRPHPGQTTAANNLYKFLSNSKLLNDGNDF-RQ
jgi|Aspni7|1126350 262 EAHQLAVLAQAVTALTVEAIQGSTESFHPFIAQVRPHEGQIEAAENILSLLKGSLLARGSSTTQTR
jgi|Aspni7|1081533 320 EANQLALFAQVVTAMCTEALMGTRRNFHPFISNARPHPGQKEVARNLYGILSDSKLASDS--IPEE
1B8F:A|PDBID|CHAIN|SEQUENCE 273 ADKVQDPYSLRCQPQVMGACLTQLRQAAEVLGIEANAVSDNPLVFAA--EGDVISGGNFHAEPVAM
jgi|Aspni7|1129557 325 GSLRQDRYSIRTSAQWMGPLLEDLELAYQQISVELNSVTDNPLIDTAGDNARILHGGNFQAKSITS
jgi|Aspni7|1126350 328 TGLVQDRYSLRTASQWIGPQLEDLLLADRQVQVELNSTSDNPLIDTG--SKTFYTGGNFQATSITS
jgi|Aspni7|1081533 384 VGLAQDRYAIRTTPQWIGPQIESLALATRQVECELNSTTDNPLLDTE--NGEVHHGGNYQAASITS
1B8F:A|PDBID|CHAIN|SEQUENCE 337 AADNLALAIAEIGSLSERRISLMMDKHMS-QLPPFLVENG-GVNSGFMIAQVTAAALASENKALSH
jgi|Aspni7|1129557 391 AMEKTRQAVQSMGRMLYAQCTELIDSSKNNGLPPNLSADEPSLSFTFKGVDIMIAALQSELGFLAN
jgi|Aspni7|1126350 392 AMEKTRLALQMFGKMLFVQCNEMIDPNLNNGLPTNLVADDPSLSFTMKGVDINMAAYMSELAYLAN
jgi|Aspni7|1081533 448 AMEKVMTCMQMIGKMIFAQCSELLNPMFSKGLPPNLCADDPNLSFAFKGIDINMASYMSELAYLAH
1B8F:A|PDBID|CHAIN|SEQUENCE 401 PHSVDSLPTSANQEDHVSMAPAAGKRLWEMAENTRGVLAIEWLGACQGLDLRKGLKTSAKLEKARQ
jgi|Aspni7|1129557 457 PIGSHVQTAEMGNQALNSLALISSRYTLDAVAVLSQMAAAHLVATCQALDLRVINIQFMR---T--
jgi|Aspni7|1126350 458 PVSSHVQTAEMQNQALNSLAFVSARYTMKAVDIVSMMGACALYVACQALDLRVLQLRFFQ---R--
jgi|Aspni7|1081533 514 PVSNFVQSAEMHNQSINSLALLTARYAGDTVEILSLMMATHIYVLCQALDLRTLQLEFEE---S--
1B8F:A|PDBID|CHAIN|SEQUENCE 467 ALRSEVAHYDRDRFFAPDIEKA-V--------EL--------------------------LAKGSL
jgi|Aspni7|1129557 518 -LEQDFMKLFGDSFQVNAVPFKNILAISKEAWDAFAKQVEQLQSVDSCSRFHQAARAILPIFIMA-
jgi|Aspni7|1126350 519 -VQGVAKEIAHG-AFGKVLEPFEIDQVADHLSEAIQNSWPSTSRLDLRDRCKRVAEMFIPVLFGAL
jgi|Aspni7|1081533 575 -AKPSVDETTRV-MCMDLVPEKRAAYIKVKIWNVLMQHWAKNSNLGLASRAATSTTHA----VGTL
1B8F:A|PDBID|CHAIN|SEQUENCE 498 TGLLPAGVLPS-------L---
jgi|Aspni7|1129557 582 ---LPQS------AASPAIIQSWIDRLGSQATETYRKTHEAYCKCPDATPYLGEASRKMYTHVRQN
jgi|Aspni7|1126350 583 LQMIPQNRQTSDLLT---AISACKMISVFKLEGVYREVFAEFCTSQPTADFLGTGTKEIYTFIRHD
jgi|Aspni7|1081533 635 INLITDECDESKIGEIPGKVLLWQKEVKSKLCDEYEMTREKYFLDPSTPDYLCTSCIKLYNYVRKT
1B8F:A|PDBID|CHAIN|SEQUENCE --
jgi|Aspni7|1129557 639 LKLPFIVEGTLWRPSDSAKTPDGEATPPPLPTMGDVVGIAYDAIRRGSLYTVAVECIRDVEQ-DMG
jgi|Aspni7|1126350 646 LRVPFHQ-GFVEHPSASQTDLPETINGRVKKTVGGWISVVYEALRNGTLSGTILNSFRQ*------
jgi|Aspni7|1081533 701 LEVPMHK-GVVDHPTFL----PPHTIPSEKQTIGSLISTIYVDLREGDLMHTLKGCFEVPLVGSVG
1B8F:A|PDBID|CHAIN|SEQUENCE -----------------
jgi|Aspni7|1129557 704 KYPHCLVNYPTCASRT*
jgi|Aspni7|1126350 -----------------
jgi|Aspni7|1081533 762 KDDEE*-----------
