# Supplementary material for: A community-driven reconstruction of the Aspergillus niger metabolic network
Source: Fungal Biol Biotechnol. 2018 Sep 26;5:16. doi: 10.1186/s40694-018-0060-7 (PMC6158834; doi:10.1186/s40694-018-0060-7)
Supplement: Supplementary file 8 — Additional file 8. Changes in expression of genes belonging to different pathways. The change of gene expression in the CBS513.88 model. [file 40694_2018_60_MOESM8_ESM.pdf]

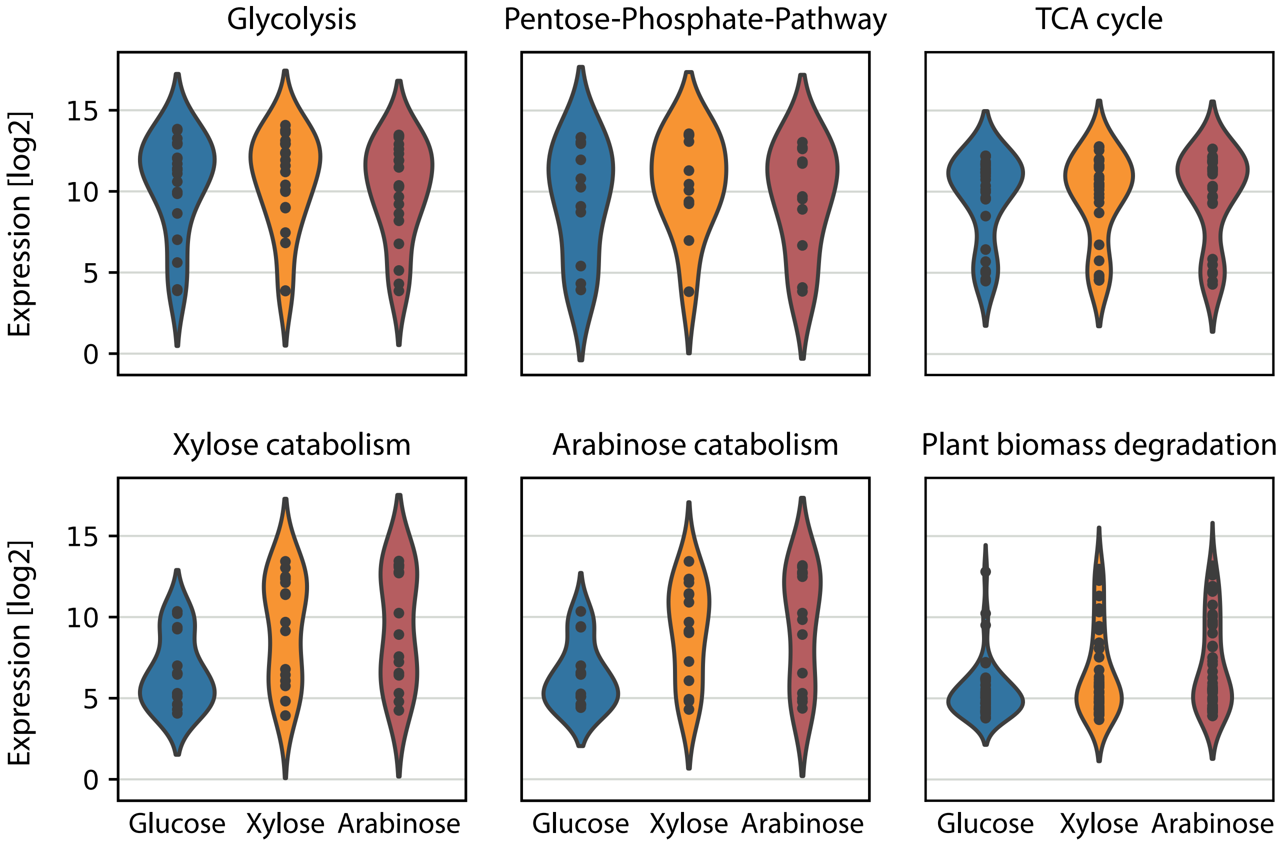
Glycolysis
Pentose-Phosphate-Pathway
TCA cycle
Xylose catabolism
Arabinose catabolism
Plant biomass degradation
Expression [log2]
Expression [log2]
0
5
10
15
Glucose
Xylose
Arabinose
